# Surveillance and control of rabies in La Reunion, Mayotte, and Madagascar

**DOI:** 10.1186/1297-9716-44-77

**Published:** 2013-09-09

**Authors:** Soa Fy Andriamandimby, Jean-Michel Héraud, Ravo Ramiandrasoa, Maherisoa Ratsitorahina, Jhon H Rasambainarivo, Laurent Dacheux, Anthony Lepelletier, Steven M Goodman, Jean-Marc Reynes, Hervé Bourhy

**Affiliations:** 1 National Laboratory for Rabies, Virology Unit, Route de l’Institut Pasteur, Institut Pasteur de Madagascar, BP 1274, 101 Antananarivo, Madagascar; 2 Centre Antirabique, Route de l’Institut Pasteur, Institut Pasteur de Madagascar, BP 1274, 101 Antananarivo, Madagascar; 3 Epidemiology Unit, Route de l’Institut Pasteur, Institut Pasteur de Madagascar, BP 1274, 101 Antananarivo, Madagascar; 4 Faculty of Medicine, Department of Veterinary, University of Antananarivo, Ankatso, 101 Antananarivo, Madagascar; 5 Lyssavirus Dynamics and Host Adaptation Unit, National Reference Centre for Rabies, WHO Collaborating Centre for Reference and Research on Rabies, Institut Pasteur, 75724 Paris, France; 6 Field Museum of Natural History, 1400 South Lake Shore Drive, Chicago 60605, IL, USA; 7 Association Vahatra, BP 3972 101 Antananarivo, Madagascar; 8 Unité de Biologie des Infections Virales Emergentes, Institut Pasteur, 21 avenue Tony Garnier, 69365 Lyon,France

## Abstract

Mayotte and La Reunion islands are currently free of animal rabies and surveillance is performed by the French Human and Veterinary Public Health Services. However, dog rabies is still enzootic in Madagascar with 4 to 10 confirmed human cases each year. The number of antirabies medical centres in Madagascar is still scarce to provide easy access to the local population for post-exposure rabies prophylaxis. Furthermore, stray dog populations are considerable and attempts to control rabies by mass campaigns of dog vaccination have not received sufficient attention from the national health authorities. To address these challenges, an expanded program to control rabies needs to be initiated by the Malagasy authorities.

## Table of contents

1. Introduction

2. Rabies in Mayotte and La Reunion

3. Rabies in Madagascar

3.1. Introduction

3.1. Laboratory surveillance of rabies

3.2. Diagnostic methods

3.2. Human rabies prophylaxis

3.1. Epidemiological data

3.2. Animal rabies

3.2. Human cases

3.2. Post-exposure prophylaxis of rabies

3.1. Virological aspects of rabies on Madagascar

3.1. Species involved in rabies epidemiology

3.2. Dog ecology

3.2. Bat ecology

4. Conclusions

5. Competing interests

6. Authors’ contributions

7. Acknowledgements

8. References

## 1. Introduction

Rabies is a lethal form of encephalitis. It is induced by neurotropic viruses of the genus *Lyssavirus*. It is a zoonotic infection mainly transmitted by the saliva of infected animals. Whereas extensive efforts in developed countries have largely controlled dog (America and Europe) and fox (Europe) rabies, dog rabies remains enzootic in many regions of the world. Rabies prevention methods have been known since the period of Louis Pasteur and have subsequently evolved to effective schedules of post-exposure prophylaxis (PEP) [1]. About 15 million people require PEP every year. However, the estimated number of human rabies deaths remains high worldwide at approximately 55 000 deaths per year [2]. Over 95% of these human rabies examples are concentrated in Asia and Africa, and dogs are responsible for the transmission in 99% of these cases. Control programs targeting dogs have been shown to effectively reduce the risk of rabies virus transmission to humans but their design and implementation still poses considerable challenges to governments of developing countries [3].

The current rabies situation on islands in the western Indian Ocean is diverse. Some countries such as the Seychelles and Mauritius are considered rabies free. On the contrary, rabies is enzootic in Madagascar. This present paper describes the surveillance system of rabies currently in place in Madagascar and the collated epidemiological data for that country and two French islands, Mayotte and La Reunion, which are located close to Madagascar. In accordance to the “One Health” concept, both veterinary and human medical aspects are covered and potential improvements, when appropriate, are discussed. For human cases, written informed consent was obtained from the patient’s guardian/parent/next of kin for the publication of this report.

## 2. Rabies in Mayotte and La Reunion

La Reunion is an island located approximately 750 km east of Madagascar. Its total surface area is 2512 km^2^ with a population of about 800 000 inhabitants. Mayotte, part of the Comoros Archipelago, consists of a main island, Grande-Terre (or Mahoré), a smaller island, Petite-Terre (or Pamanzi), and several nearby islets. It is located in the northern Mozambique Channel. Its total surface area is 374 km^2^ with an estimated population of 194 000. Administratively, La Reunion and Mayotte are two overseas departments of France.

In France, rabies is a “notifiable disease”, which means that by law one must report rabies cases to the governmental authorities when diagnosed. The epidemiological surveillance of rabies in animals and humans is performed by the Ministry of Agriculture and by the Ministry of Health and by their respective agencies, the Institut de Veille Sanitaire [4] and the Agence Nationale de Sécurité Sanitaire de l'Alimentation, de l'Environnement et du Travail [5]. The primary health-care management of patients seeking PEP is delivered through an official national network of Antirabies Medical Centres (ARMC), that are distributed throughout the country [6,7]. One of these centres is located in Saint Denis on La Reunion. No ARMC exists on Mayotte and patients seeking PEP are obliged to go to Saint Denis or vaccines have to be air-shipped to Mayotte. PEP is predominantly administered according to the Zagreb schedule [1]. Clinicians conduct a risk assessment for each exposed patient, and decide to administer PEP according to the general recommendations, epidemiological data and nature of the bite. The French network for rabies prophylaxis provides exhaustive national data collected by ARMC and analyzed by the National Reference Centre for Rabies, (NRCR) [8]. Rabies diagnosis in humans is based on specimens collected *intra*-*vitam* (mainly saliva and skin biopsy) and specimens collected *post*-*mortem* (skin and brain biopsies). These samples are analysed by RT-hnPCR [9]. Terminal brain biopsies are analysed by the fluorescent antibody test (FAT) and by the rapid tissue culture isolation test (RTCIT). Both techniques are recommended by WHO.

French veterinary authorities are in charge of the surveillance of animal rabies. Each animal responsible for human exposure is confined under veterinary surveillance. In cases when the animal dies or for some other reasons, diagnostic laboratory tests are conducted at the NRCR, Institut Pasteur, Paris, France, where rabies diagnosis in animals is based on the FAT and RTCIT techniques [10].

Both Mayotte and La Reunion are considered free of rabies in non-flying animals according to the criteria of the World Organization for Animal Health (OIE). The surveillance of rabies on these islands continues today. From 2006 to 2011, one animal sample from Mayotte and 16 from La Reunion were submitted to the NRCR for rabies diagnosis (Table 1). All of them tested negative. The last human case reported in these departments was in 1996 on La Reunion. The patient was a three-year-old boy living in La Reunion. He was bitten by a suspected dog during his holidays in Madagascar and did not receive any PEP.

**Table 1:** **Submission of specimens from Mayotte**, **La Reunion and Madagascar for laboratory diagnosis of rabies from 2006 to 2011**

**Species**	**Origin**	**Number of samples**	**Tested positive (%)**
Dog	La Reunion	10	0
Dog	Mayotte	4	0
Cat	La Reunion	1	0
Mice	La Reunion	2	0
Human	Madagascar	22	20 (90.9)
Dog	Madagascar	373	195 (52.3)
Cat	Madagascar	52	14 (26.9)
Cattle	Madagascar	25	20 (80.0)
Pig	Madagascar	3	2 (66.6)
Rabbit	Madagascar	2	0
Lemur	Madagascar	9	0
Rat	Madagascar	5	0
Fossa*	Madagascar	1	0
Total		509	251 (49.3)

* = (*Cryptoprocta ferox* (Order Carnivore, family Eupleridae).

## 3. Rabies in Madagascar

### 3.1. Introduction

Madagascar, located in the southwestern Indian Ocean and about 400 km to the east of the African coast, is the fourth largest island in the world. The country is subdivided into 22 administrative areas, 111 districts and 2800 municipalities. In 2009, the human population was approximately 20.6 million inhabitants. Madagascar is one of the poorest countries in the world with a health system that is hardly efficient to monitor and prevent disease outbreaks, and the medical monitoring system is largely based on districts. The medical personal/population ratio approaches a satisfactory situation in urban areas, but the rural areas remain notably underprivileged.

The health sector is co-funded by the government (32%), donors (36%) and private sector (32%). The provision of health services is coordinated by the Secrétaire Général de la Santé, who is assisted by the Directeur Général de la Santé. They both operate under the Département des Urgences et de la Lutte Contre les Maladies Négligées (DULMN) and the Direction de la Veille Sanitaire et de la Surveillance épidémiologique (DVSSE). The DULMN and DVSSE coordinate all activities related to disease surveillance and response. Since 1996 and in accordance with a WHO resolution known as AFR/RC43/R7, the Integrated Diseases Surveillance and Response (IDSR) system is operational on Madagascar and human rabies is one of the main diseases under surveillance.

The rabies virus has circulated in Madagascar at least since the 19th century. The first reported death from rabies occurred in 1896 and two years later the Institut Pasteur de Madagascar (IPM) was established. The first rabies post-exposure treatment using rabies vaccine was implemented in 1902. Since that period, several reports have described different aspects of rabies on the island [11-15]. The last one, covering the period from 1982 to 2010, indicated that rabies was prevalent in five provinces and that dogs were the primary vector of the virus [14,15].

Since 1963, several law texts were signed by national authorities to address measures to fight rabies [16] (Ministère de l'Agriculture de l'Elevage et de la Pêche: Arrêté N°3482/99 du 12 avril 1999 fixant les mesures de lutte contre la rage, unpublished; Ministère de l'Agriculture de l'Elevage et de la Pêche: Arrêté N°3483/99 du 12 avril 1999 relatif à l’observation des animaux mordeurs, unpublished) or to regulate feral dogs [17]). In 1978, another text was signed for the creation of an inter-ministerial committee against rabies (Ministère de l'Agriculture de l'Elevage et de la Pêche: Arrêté N°3894/78/PM du 24 aout 1978 portant création d’un comité interministériel de lutte contre la rage, unpublished). Despite the existence of these official texts, their application, both in urban and rural areas, is frequently not effective. No formal coordination exists between the Ministry of Public Health and the Veterinary Services.

### 3.2. Laboratory surveillance of rabies

In Madagascar, there is only one authorised national laboratory for the diagnosis of rabies (NRL), which is located at the IPM. Animal specimens are transmitted by veterinarians, animal health officers, animal owners or exposed individuals. Further, hospital staffs send specimens collected from humans suspected to have died from rabies to the IPM. Rabies surveillance, and notification, was a national program initiated and approved by the ministries of health and Ethics committee of Madagascar (FWA00016900). Before taking each specimen, physicians explained the purpose of the notification. Patients’ relatives were then free to refuse sample collection.

#### 3.2.1. Diagnostic methods

At the IPM, rabies diagnosis is routinely performed by rabies antigen detection using FAT, which is generally performed in a post-mortem manner on brain tissue of suspected animals or humans. To confirm negative results obtained by FAT in animal samples suspected for rabies, rabies virus isolation is performed systematically in cell culture (murine neuroblastoma cell line). Human cases are post-mortem diagnosed using skin biopsies taken from the nape of the neck. Detection of rabies virus RNA is performed using RT-hn PCR [9].

For epidemiological surveys, specimens are also collected from bats. The presence of lyssavirus RNA was investigated by RT-hn PCR [9,18]. In parallel, virus neutralisation assay is used to detect antibodies against different species of lyssavirus. To extend the potential spectrum of detection of this survey and to search for the circulation of any presently uncharacterised lyssaviruses, neutralisation assays were performed using in parallel 6 different species of lyssaviruses: rabies virus (RABV), Lagos Bat virus (LBV), European Bat Lyssavirus type 1 (EBLV-1) and 2 (EBLV-2), Mokola virus and Australian Bat Lyssavirus [19].

#### 3.2.2. Human rabies prophylaxis

**Figure 1:**
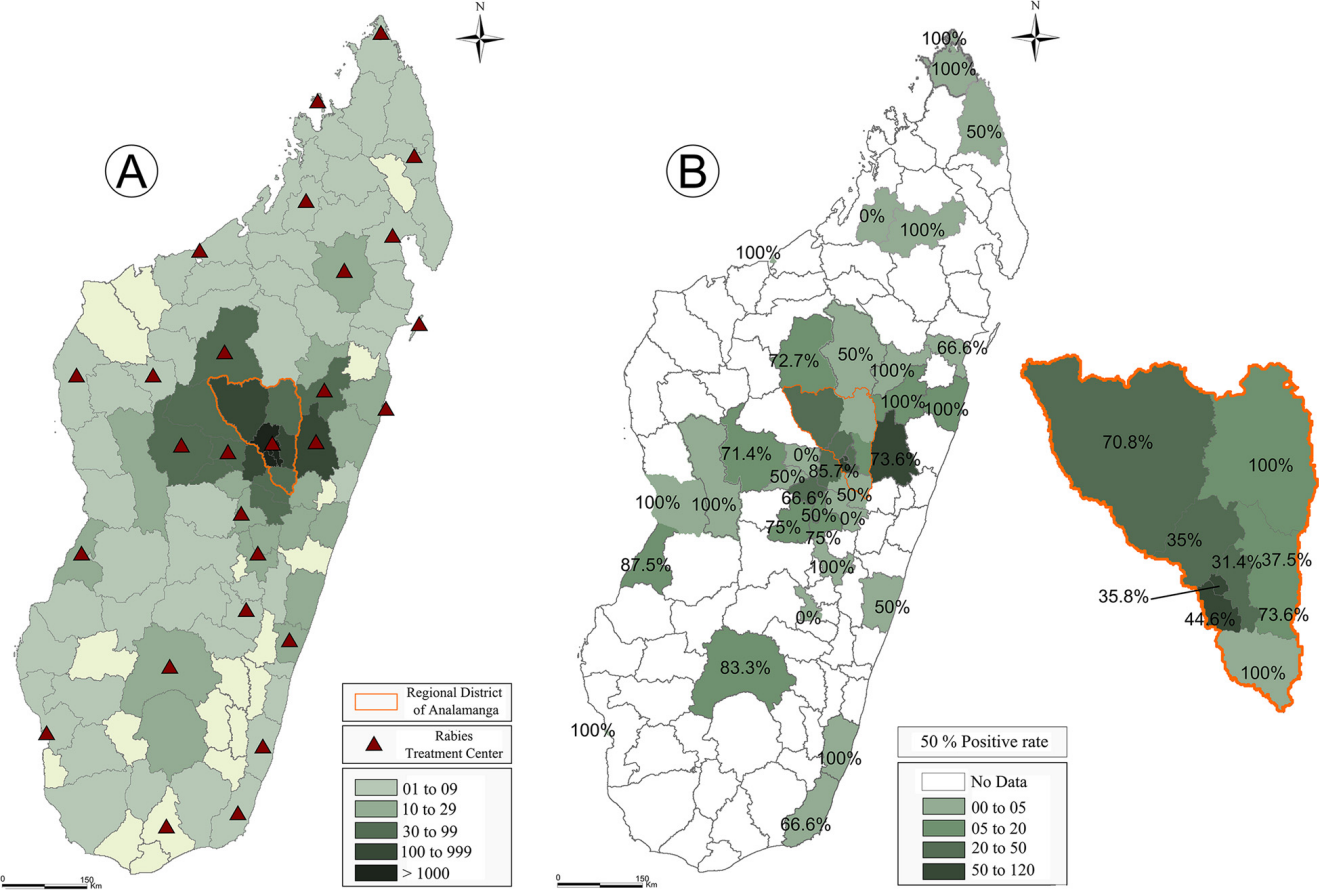
**PEP and rabies positive animal samples in Madagascar. (A)** Geographical origin of patients receiving PEP at the antirabies medical centre (ARMC) of the Institut Pasteur de Madagascar (IPM) in Antananarivo (2006–2011). Green gradient colour represents the number of bite cases that consult at IPM from different districts. Red triangles represent the ARMC located in each administrative region of Madagascar. **(B)** Geographical distribution of samples received at the National Laboratory Reference for rabies diagnostic. Madagascar, 2006–2011.

Until March 2006, the ARMC at the IPM used Nerve-Tissue-derived Vaccine (NTV) produced by the Institut Pasteur in Alger (Algeria). During this period, this low-cost vaccine could be sent on demand to all health centres in the different health districts (*n* = 111). The notably more expensive Purified Vero Cells Rabies Vaccine (PVRV), the only one in use in Madagascar since 2006, could not be provided to all health centres for economic reasons. Subsequently, the total number of ARMC that could perform PEP was reduced to 27 (Figure 1a). Each ARMC is the unique source for PEP in the corresponding administrative region. PVRV is provided by IPM but each ARMC controls its own vaccine stock. PEP is provided free of charge and administered by the use of the modified Red-Thai Cross protocol (i.e. 2 intradermal injections of 0.1 mL at two sites, deltoid and thigh, on days 0, 3, 7 and 28) [1,20]. In case of severe exposure, purified rabies immune globulins (RIG) (Equirab®, Bharat serums and Vaccines Limited) are administered to the patients. RIG are only delivered by the ARMC at the IPM and are given free of charge. PEP is discontinued in case the suspected animal is found negative via laboratory diagnosis or when it is still alive after a 10-day observation period starting from the date of the bite (or the exposure) [1].

### 3.3. Epidemiological data

#### 3.3.1. Animal rabies

Between 2006 and 2011, NRL received 470 animal specimens and 231 (49.1%) tested positive (Table 1). The majority of specimens originated from the urban area surrounding the capital, Antananarivo (Figure 1b). Dogs represented more than 75% of specimens. Among the positive specimens, 195 (78%) were from dogs. PEP was administered to patients for 89 animal bites that were confirmed positive and for the remaining (30 cases), PEP was not initiated. Rabies circulation has been documented in 38 of the 111 districts (Figure 1b).

**Figure 2:**
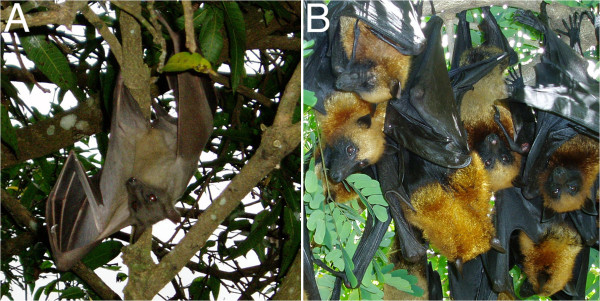
**Bat species showing serological evidence of the circulation of lyssaviruses in Madagascar: (A) *Eidolon dupreanum *and (B) *Pteropus rufus.****Eidolon dupreanum* and *Pteropus rufus* (or Madagascar fruit bats) are species of megabats in the Pteropodidae family and are endemic to Madagascar.

As part of the research program at the IPM, detection of lyssavirus was investigated in insectivorous and frugivorous bats, including two areas close to Antananarivo. No lyssavirus RNA was detected in the different individuals tested. However, 18 Malagasy fruit bats were tested positive for the detection of antibodies against LBV (12 *Eidolon dupreanum*) or against both EBLV-1 and LBV (five *E*. *dupreanum* and one *Pteropus rufus*) (Figure 2) [15]. These results are in favour of the circulation of some lyssaviruses antigenically related to EBLV-1 and/or LBV in Malagasy fruit bats of the family Pteropodidae.

#### 3.3.2. Human cases

Over the course of the past six years, 22 human specimens were received at the NRL and 20 tested positive. During 2011, NRL received 11 specimens, all positives, and seven of which were from Toamasina District on the east coast. None of these patients received PEP with the exception of one who started PEP late, 10 days after the suspected bite.

Besides these confirmed laboratory cases, from different data sources, 10 clinically suspected cases of rabies in humans were reported in 2011 without further confirmation. Cultural barriers and lack of information available for local people are among the main problems explaining the low rate of laboratory confirmation of human cases. This highlights the significant under-reporting of human rabies on the island.

#### 3.3.3. Post-exposure prophylaxis of rabies

**Figure 3:**
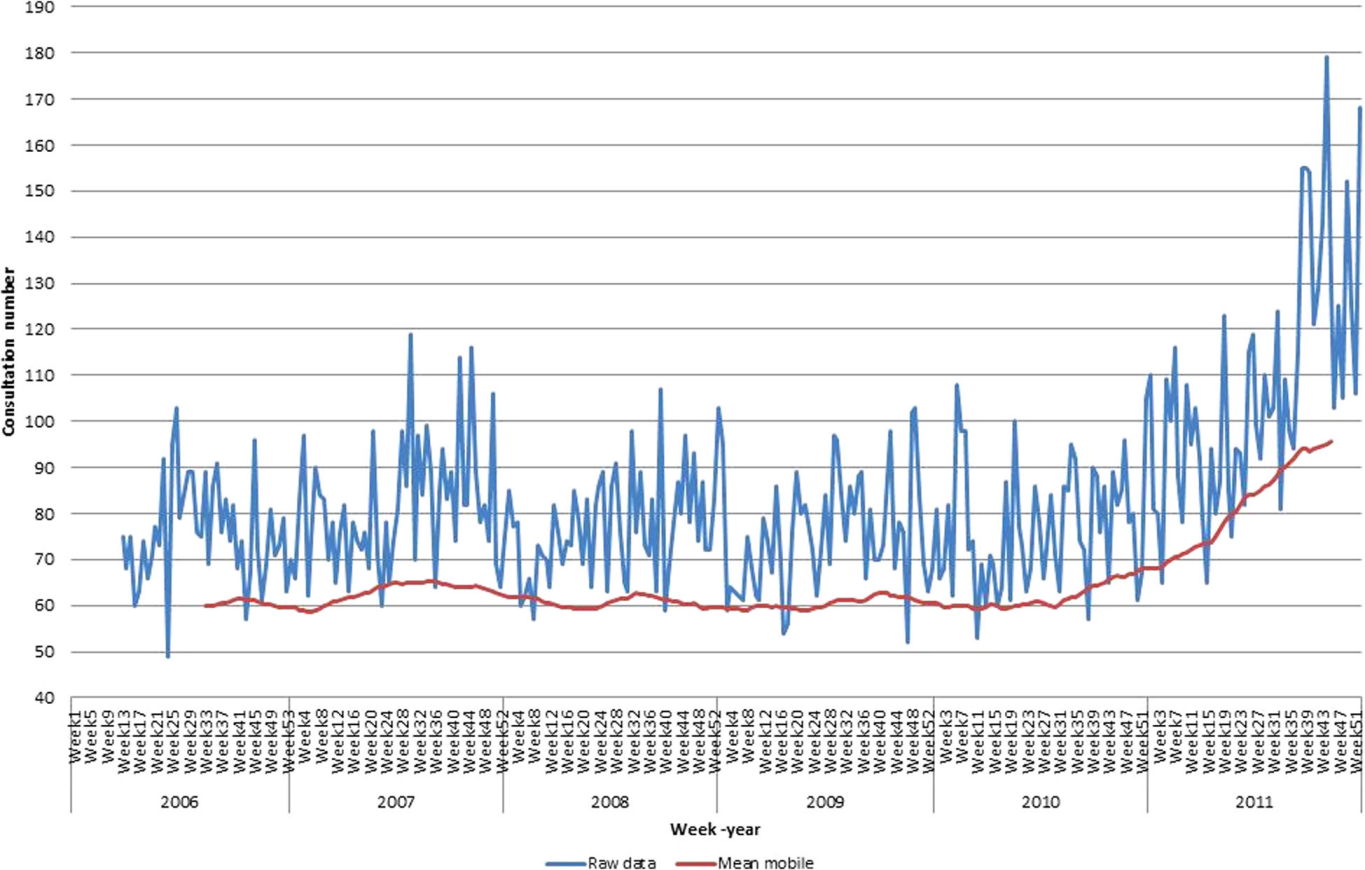
**Pattern of consultations at antirabies medical centre (ARMC) – Institut Pasteur de Madagascar (IPM), Antananarivo, 2006–2011.** Mean of weekly visit counts at ARMC (IPM) for 5 years. Weekly visit time series plots (number) (blue-curve) and the moving average (red-curve) for weekly visit counts.

Since the use of PVRV in mid-March 2006 until 31 December 2011, 24 946 patients visited the ARMC at the IPM, of which 97.2% (*n* = 24 299) received PEP. Males represented 54.3% (*n* = 13 556) of the cases and ranged in age from one to 97 years (median = 18 years). Children under 15 years old represented 40.5% (*n* = 10 107) of the consultants. The number of consultations showed little fluctuation over the past five years, with a mean incidence rate of three per 1000 inhabitants (Figure 3), with the exception of 2011, when an increase (49.3%) in the number of consultations was recorded. This growth can be explained partly by a rising number of post-exposure treatments associated with an information campaign of governmental authorities for people in contact with dogs that have tested positive in a laboratory context. The rabies day celebrated in 2011 in Madagascar resulted in an increase in the number of consultations. A large proportion of the patients (28.1%) did not complete their PEP regime. Dogs were responsible for 91.3% (*n* = 22 770) of the bite cases. Among the patients receiving PEP, 65% were bitten by domestic dogs for which surveillance was effective and completed in only 61% of the cases. Stray dogs bit a significant proportion of the patients (*n* = 6552 (28.7%)).

The ARMC of the IPM is responsible for animal bite cases from the Analamanga Region, as well as handling cases coming from other areas of Madagascar (Figure 1a). Data are not available from the other ARMC. Nevertheless, considering the number of vials sent to the different ARMC (59 401) and given that three vials are used per patient on average, if all were administered, this would be equivalent to the treatment of about 19 800 patients per year.

### 3.4. Virological aspects of rabies on Madagascar

**Figure 4:**
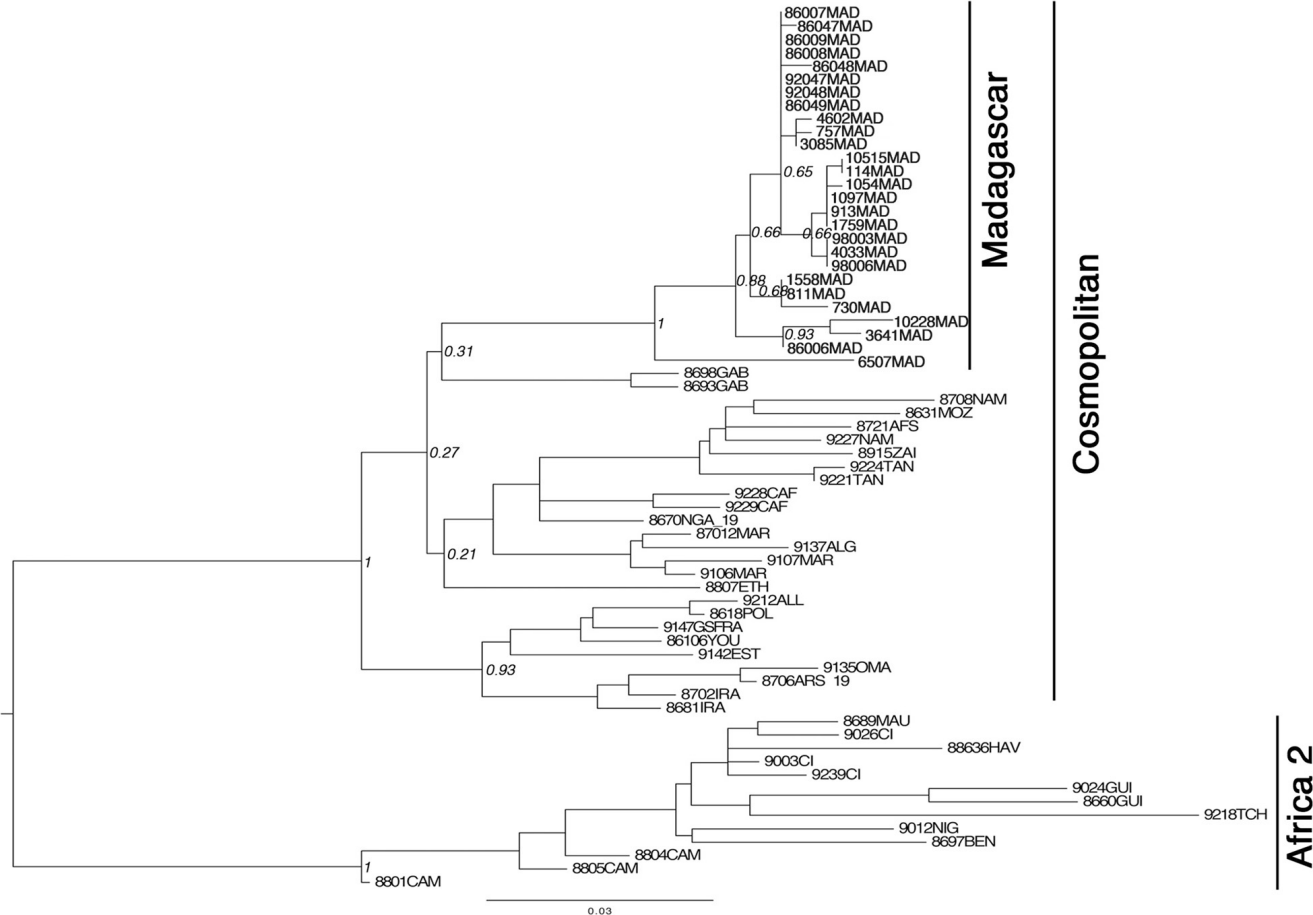
**Maximum-likelihood phylogenetic tree of 66 partial N gene sequences (Positions 127**–**629 nucleotides of the rabies virus genome [**[30]**]) showing phylogenetic relationships of Malagasy isolates with other isolates originating from Africa**. The phylogenetic trees was estimated using a maximum-likelihood (ML) method under the general time-reversible (GTR) model of nucleotide substitution, with the rate of each substitution type estimated from the data using PHYML 3.0 [31]. The ML base frequencies were also estimated from the data, as were the proportion of invariable sites (I) and a gamma distribution of rate variation among sites (Γ) with four rate categories. Tree topology was estimated using the best estimates among simultaneous Nearest Neighbour Interchanges and the approach relying on subtree pruning and regrafting. The support of the data for each internal branch of the phylogeny was estimated using non-parametric bootstrap. Bootstrap support of nodes discussed in the text is indicated. The tree is rooted between the Africa 2 and the Cosmopolitan lineages. The generated sequences have been deposited in GenBank under accession numbers KC787050 to KC787074, DQ420623 and U22854.

The partial nucleoprotein gene (502 nucleotides) of a subset of selected isolates of dead-end host (cattle, pigs and humans) was sequenced and compared with viruses isolated from dogs during different periods and from different areas [21-23] (Table 2). Phylogenetic analyses of the different viruses (*n* = 27) showed that all isolates belong to the RABV species (Figure 4) and to a subset of the cosmopolitan lineage named “Africa 1”, largely distributed in Africa [22,24]. Their phylogenetic origin is quite different from the viruses circulating in countries of Africa bordering the eastern part of the Mozambique Channel (Tanzania, Mozambique and South Africa) [25-29] as such, excluding a recent importation of rabies from these countries to Madagascar. The most probable hypothesis is an introduction of rabies to Madagascar during the colonisation period as observed in other former French colonies in Africa [23,24]. The topology of the tree and the bootstrap values also show some geographical clustering. Although this needs to be further confirmed with a larger number of isolates and with longer sequences, three virus clusters are identified in three different geographical areas: the north (isolate 6507MAD), southwest (isolates 86006MAD, 3641MAD, 10228MAD) and the centre and the west coast (isolates 10515MAD, 4602MAD, 3085MAD, 730MAD, 811MAD, 1558MAD, 114MAD, 1054MAD, 07057MAD, 913MAD, 1097MAD, 1759MAD, 4033MAD, 98003MAD, 98006MAD, 92047MAD, 92048MAD, 86007MAD, 86008MAD, 86009MAD, 86047MAD, 86048MAD, 8604MAD9). The viruses circulating in the north and on the west coast are also found in Antananarivo. This diversity probably reflects translocation of infected animals between the capital city (1.6 million inhabitants) and the countryside. This transportation probably occurs along the roads due to human intervention as reported in North Africa [24].

**Table 2:** Origin and phylogeography of rabies virus in Madagascar from 1984 to 2011

**Sequence ID**	**Collection year**	**City**	**Host**	**Accession N°**
10515MAD_2011	2011	Antananarivo	Dog	KC787050
10228MAD_2009	2009	Taolagnaro	Dog	KC787051
4602MAD_2009	2009	Toamasina*	Human	KC787052
114MAD_2008	2008	Faratsiho	Cattle	KC787053
3085MAD_2008	2008	Ihosy	Cattle	KC787054
3641MAD_2008	2008	Ihosy	Swine	KC787055
6507MAD_2008	2008	Antsiranana	Cattle	KC787056
730MAD_2007	2007	Antananarivo	Human	KC787057
757MAD_2007	2007	Antananarivo	Human	KC787058
1054MAD_2007	2007	Maevatanana	Dog	KC787059
811MAD_2006	2006	Ambatondrazaka	Cattle	KC787060
913MAD_2006	2006	Maevatanana	Cattle	KC787061
1097MAD_2006	2006	Moramanga	Cattle	KC787062
1558MAD_2006	2006	Tsaratanana	Cattle	KC787063
1759MAD_2006	2006	Maevatanana	Swine	KC787064
4033MAD_2004	2004	Antananarivo	Human	DQ420623
98003MAD_1998	1998	Antananarivo	Human	KC787065
98006MAD_1998	1998	Antananarivo	Human	KC787066
92048MAD_1991	1991	Antananarivo	Human	KC787067
92047MAD_1990	1990	Antananarivo	Dog	U22854
86047MAD_1986	1986	Antananarivo	Dog	KC787068
86048MAD_1986	1986	Antananarivo	Dog	KC787069
86049MAD_1986	1986	Mahitsy	Dog	KC787070
86007MAD_1985	1985	Antananarivo	Cat	KC787071
86008MAD_1985	1985	Antananarivo	Cat	KC787072
86009MAD_1985	1985	Antananarivo	Dog	KC787073
86006MAD_1984	1984	Morondava	Dog	KC787074

* = Associated with the bite of a *tenrec ecaudatus* (Order Afrosorocida, family Tenrecidae).

Since no lyssavirus has been isolated on the island from bats, little can be mentioned about local diversity, and the epidemiological aspects mentioned above for the seropositive results observed in Malagasy fruit bats.

### 3.5. Species involved in rabies epidemiology

#### 3.5.1. Dog ecology

There is no available census of the number of dogs living in Madagascar. The only recent data available concerns a study in Antananarivo. This work indicated that dogs are abundant in the capital as compared to other major cities on the island, less than 10% of the animals are vaccinated against rabies, and more than 10% of the estimated 500 000 are strays [32]. No information is available on the movements of these dogs, population dynamics or on the introduction of animals from different populations.

#### 3.5.2. Bat ecology

Fourty-five species of bats are known from Madagascar, of which 76% are endemic to the island [33-36]. Amongst these, 42 are insectivorous and three frugivorous. A number of species can be found roosting in human-occupied or abandoned buildings, including hospitals and schools. Presumably, these animals have a greater chance of being in contact with humans than those living exclusively in the wild. In certain cases, roost sites can be occupied by many hundreds of bats and include those of the family Molossidae (principally *Chaerephon atsinanana* (formerly *C*. *pumilus* or *Tadarida pumila*), *C*. *leucogaster*, *Mops leucostigma*, *M*. *midas*, *Mormopterus jugularis*) and the family Vespertilionidae (*Neoromicia matroka* (formerly *Eptesicus matroka*) and *Pipistrellus raceyi*). Little data are available on the fidelity of individual bats to a given roost site, but based on phylogeographic studies of members of the family Molossidae, certain species show considerable dispersal capacity [37,38]. The epidemiology and mode of transmission of the suspected bat lyssavirus responsible for the positive serologic reaction in *Eidolon dupreanum* and *Pteropus rufus* (Figure 2) remains unknown and should be a focus of research. Both of these bats feed exclusively on fruit, pollen and nectar and live in colonies; the former making its day roost sites in caves and rock crevasses and the latter suspended from trees. These two species, along with the third species of fruit bat found on Madagascar, *Rousettus madagascariensis*, can be found at night feeding in the same fruit trees. People visiting these trees and feeding on the fruits risk the possibility of coming in contact with fruit bat saliva.

## 4. Conclusions

Mayotte and La Reunion are free of animal rabies. However, dog rabies is still enzootic in Madagascar. Serological evidence also suggests that other species of lyssaviruses may circulate in at least two Malagasy fruit bat species. The advancement of programs to monitor rabies in Madagascar suffers from a lack of public education and a poorly functional and inefficient system for routine diagnosis of suspected cases (animal or human). The number and the origin of samples received at the NRL are insufficient to address different aspects of the disease in Madagascar (i.e. prevalence in animals, systematic sampling of suspected human cases, etc.). Access to PEP for people living in some rural areas is limited and numerous other cities and villages are requesting for the establishment of a local ARMC. Nevertheless, without more accurate information on the local epidemiology of the virus, as well as for economic reasons (cost of vaccine, need to maintain refrigeration, etc.), the expansion of the ARMC network remains difficult.

Previous campaigns to reduce stray dog populations were ineffective for different reasons. This method is costly for a country like Madagascar. Stray dogs are increasing in number due to the expanding food sources (e.g., sites with accessible trash, open slaughterhouses) and for cultural reason (no contraceptive measures for owned pets, expanding interest in dogs as house pets, etc.). To address these challenges, an expanded program to control rabies needs to be initiated by national authorities and advocacy programs should be initiated with the different stakeholders.

The chance of being able to control rabies on Madagascar is high, as it is an island, and the elimination of rabies and its sustainability need to coincide with limiting the introduction of rabid animals from neighbouring enzootic countries. Therefore, an eradication program needs to be first promoted at a pilot scale, in order to test and validate the tools used for canine vaccination and population management, education and information to the general population and public health professionals. If successful, it can then be extended to an island-wide scale.

## 5. Competing interests

The authors declare that they have no competing interests.

## 6. Authors’ contributions

SFA: wrote the paper, carried out statistical analysis. JMH: wrote the paper and coordinate rabies surveillance in Madagascar. RR: compiled all human cases data. MR: compiled and analysed dog data ecology. JHR: helped to draft the paper. LD: carried out molecular and phylogenetic analyses of rabies strains. AL: carried out molecular and phylogenetic analyses of rabies strains. SMG: helped to draft the paper and carried out bat studies. JMR: helped to draft the paper and carried out all bat studies. HB: wrote and coordinate the paper and compiled all data from La Reunion, Mayotte and Madagascar. All authors read and approved the final manuscript.

## References

[B1] WHO Rabies vaccines: WHO position paper--recommendations Vaccine 2010 28 7140 7142 20831913 10.1016/j.vaccine.2010.08.082

[B2] Knobel DL Cleaveland S Coleman PG Fevre EM Meltzer MI Miranda ME Shaw A Zinsstag J Meslin FX Re-evaluating the burden of rabies in Africa and Asia Bull World Health Organ 2005 83 360 368 15976877 PMC2626230

[B3] Lembo T Attlan M Bourhy H Cleaveland S Costa P de Balogh K Dodet B Fooks AR Hiby E Leanes F Meslin FX Miranda ME Müller T Nel LH Rupprecht CE Tordo N Tumpey A Wandeler A Briggs DJ Renewed global partnerships and redesigned roadmaps for rabies prevention and control Vet Med Int 2011 2011 923149 21776359 10.4061/2011/923149 PMC3135331

[B4] The Institut de Veille Sanitaire (INVS) http://www.invs.sante.fr/fr/Dossiers-thematiques/Maladies-infectieuses/Zoonoses/Rage. (Last access May 7, 2013)

[B5] The Agence Nationale de Sécurité Sanitaire (ANSES) http://www.anses.fr/fr/content/la-rage. (Last access May 7, 2013)

[B6] Gautret P Ribadeau-Dumas F Parola P Brouqui P Bourhy H Risk for rabies importation from North Africa Emerg Infect Dis 2011 17 2187 2193 10.3201/eid1712.110300 22185767 PMC3311213

[B7] Lardon Z Watier L Brunet A Bernede C Goudal M Dacheux L Rotivel Y Guillemot D Bourhy H Imported episodic rabies increases patient demand for and physician delivery of antirabies prophylaxis PLoS Negl Trop Dis 2010 4 e723 10.1371/journal.pntd.0000723 20582307 PMC2889823

[B8] The National Reference Centre for Rabies (NRCR) http://www.pasteur.fr/ip/easysite/pasteur/fr/sante/centres-nationaux-de-reference-et-centres-collaborateurs-de-l-oms/cnr-et-ccoms/cnr-de-la-rage/actualites-rapports

[B9] Dacheux L Reynes JM Buchy P Sivuth O Diop BM Rousset D Rathat C Jolly N Dufourcq JB Nareth C Diop S Iehlé C Rajerison R Sadorge C Bourhy H A reliable diagnosis of human rabies based on analysis of skin biopsy specimens Clin Infect Dis 2008 47 1410 1417 10.1086/592969 18937576

[B10] Bourhy H Rollin PE Vincent J Sureau P Comparative field evaluation of the fluorescent-antibody test, virus isolation from tissue culture, and enzyme immunodiagnosis for rapid laboratory diagnosis of rabies J Clin Microbiol 1989 27 519 523 2654181 10.1128/jcm.27.3.519-523.1989 PMC267350

[B11] Brygoo ESP Rabies in Madagascar from 1951 to 1958 Arch Inst Pasteur Madagascar 1960 38 61 96 (in French)

[B12] Coulanges P Rakotonirina-Randriambeloma PJ Epidemiology of rabies in Madagascar Arch Inst Pasteur Tunis 1982 59 47 74 (in French) 6758714

[B13] Mayoux A Coulanges P Human rabies in Madagascar. Observation of 79 cases from 1899 to 1968 Arch Inst Pasteur Madagascar 1969 38 125 145 (in French)

[B14] Morvan JM Rakoto-Andrianarivelo M Randriamihoatra S Roux J Situation of endemic rabies in Madagascar Arch Inst Pasteur Madagascar 1993 60 5 8 (in French) 8192541

[B15] Reynes JM Andriamandimby SF Razafitrimo GM Razainirina J Jeanmaire EM Bourhy H Heraud JM Laboratory surveillance of rabies in humans, domestic animals, and bats in madagascar from 2005 to 2010 Adv Prev Med 2011 2011 727821 21991442 10.4061/2011/727821 PMC3170745

[B16] Ministère de l'Agriculture de l'Elevage et de la Pêche Décret N°95-375 du 23 Mai 1995 portant définition et codification des mesures sanitaires à prendre contre la rage Officiel de la République Malgache du 1995 13 11 Antananarivo, Madagascar

[B17] Ministère de l'Agriculture de l'Elevage et de la Pêche Décret N°63-443 du 11 juillet 1963 rendant obligatoire l’abattage des chiens errants sur toute l’étendue du territoire de la République Malgache Journal Officiel de la République Malgache du 1963 20 07 Antananarivo, Madagascar

[B18] Dacheux L Wacharapluesadee S Hemachudha T Meslin FX Buchy P Reynes JM Bourhy H More accurate insight into the incidence of human rabies in developing countries through validated laboratory techniques PLoS Negl Trop Dis 2010 4 e765 10.1371/journal.pntd.0000765 21152054 PMC2994914

[B19] Reynes JM Molia S Audry L Hout S Ngin S Walston J Bourhy H Serologic evidence of lyssavirus infection in bats, Cambodia Emerg Infect Dis 2004 10 2231 2234 10.3201/eid1012.040459 15663870 PMC3323374

[B20] Quiambao BP Dimaano EM Ambas C Davis R Banzhoff A Malerczyk C Reducing the cost of post-exposure rabies prophylaxis: efficacy of 0.1 ml PCEC rabies vaccine administered intradermally using the Thai Red Cross post-exposure regimen in patients severely exposed to laboratory-confirmed rabid animals Vaccine 2005 23 1709 1714 10.1016/j.vaccine.2004.09.027 15705476

[B21] Bourhy H Reynes JM Dunham EJ Dacheux L Larrous F Huong VT Xu G Yan J Miranda ME Holmes EC The origin and phylogeography of dog rabies virus J Gen Virol 2008 89 2673 2681 10.1099/vir.0.2008/003913-0 18931062 PMC3326349

[B22] Kissi B Tordo N Bourhy H Genetic polymorphism in the rabies virus nucleoprotein gene Virology 1995 209 526 537 10.1006/viro.1995.1285 7778285

[B23] Talbi C Holmes EC de Benedictis P Faye O Nakoune E Gamatie D Diarra A Elmamy BO Sow A Adjogoua EV Sangare O Dundon WG Capua I Sall AA Bourhy H Evolutionary history and dynamics of dog rabies virus in western and central Africa J Gen Virol 2009 90 783 791 10.1099/vir.0.007765-0 19264663

[B24] Talbi C Lemey P Suchard MA Abdelatif E Elharrak M Nourlil J Faouzi A Echevarria JE Vazquez Moron S Rambaut A Campiz N Tatem AJ Holmes EC Bourhy H Phylodynamics and human-mediated dispersal of a zoonotic virus PLoS Pathog 2010 6 e1001166 10.1371/journal.ppat.1001166 21060816 PMC2965766

[B25] Cohen C Sartorius B Sabeta C Zulu G Paweska J Mogoswane M Sutton C Nel LH Swanepoel R Leman PA Grobbelaar AA Dyason E Blumberg L Epidemiology and molecular virus characterization of reemerging rabies, South Africa Emerg Infect Dis 2007 13 1879 1886 10.3201/eid1312.070836 18258039 PMC2874428

[B26] Davis PL Rambaut A Bourhy H Holmes EC The evolutionary dynamics of canid and mongoose rabies virus in Southern Africa Arch Virol 2007 152 1251 1258 10.1007/s00705-007-0962-9 17401615

[B27] Muleya W Namangala B Mweene A Zulu L Fandamu P Banda D Kimura T Sawa H Ishii A Molecular epidemiology and a loop-mediated isothermal amplification method for diagnosis of infection with rabies virus in Zambia Virus Res 2012 163 160 168 10.1016/j.virusres.2011.09.010 21930165

[B28] Sabeta CT Bingham J Nel LH Molecular epidemiology of canid rabies in Zimbabwe and South Africa Virus Res 2003 91 203 211 10.1016/S0168-1702(02)00272-1 12573499

[B29] Weyer J Szmyd-Potapczuk AV Blumberg LH Leman PA Markotter W Swanepoel R Paweska JT Nel LH Epidemiology of human rabies in South Africa, 1983–2007 Virus Res 2011 155 283 290 10.1016/j.virusres.2010.10.023 21036195

[B30] Ratsitorahina M Rasambainarivo JH Raharimanana S Rakotonandrasana H Andriamiarisoa MP Rakalomanana FA Richard V Dog ecology and demography in Antananarivo, 2007 BMC Vet Res 2009 5 21 10.1186/1746-6148-5-21 19486516 PMC2700795

[B31] Goodman SM [Bats from Madagascar] Margulis L: Origin of Eukaryotic Cells 1970 New Haven: Yale University Press edn. Antananarivo: Association Vahatra ed.; 2011

[B32] Goodman SM Ramasindrazana B Maminirina CP Schoeman MC Appleton B Morphological, bioacoustical, and genetic variation in *Miniopterus* bats from easter Madagascar, with the description of a new species Zootaxa 2011 2880 1 19

[B33] Goodman SM Les chauves-souris de Madagascar 2011 Antananarivo: Association Vahatra 129 pp

[B34] Goodman SM Taylor PJ Ratrimomanarivo F Hoofer S The genus *Neoromicia* (Family Vespertilionidae) in Madagascar, with the description of a new species Zootaxa 2012 3250 1 25

[B35] Ratrimomanarivo F Goodman SM Taylor PJ Melson B Lamb J Morphological and genetic variation in *Mormopterus jugularis* (Chiroptera: Molossidae) in different bioclimatic regions of Madagascar Mammalia 2009 73 110 129

[B36] Ratrimomanarivo FH Goodman SM Stanley WT Naidoo T Taylor PJ Lamb J Geographic and Phylogeographic variation in Chaerephon leucogaster (Chiroptera: Molossidae) of Madagascar and the Western Indian Ocean Islands of Mayotte and Pembafull access Acta Chiropterol 2009 11 25 52 10.3161/150811009X465677

[B37] Delmas O Holmes EC Talbi C Larrous F Dacheux L Bouchier C Bourhy H Genomic diversity and evolution of the lyssaviruses PLoS One 2008 3 e2057 10.1371/journal.pone.0002057 18446239 PMC2327259

[B38] Guindon S Gascuel O A simple, fast, and accurate algorithm to estimate large phylogenies by maximum likelihood Syst Biol 2003 52 696 704 10.1080/10635150390235520 14530136

